# Supplementary figures and images for: Genomic characterization of Francisella tularensis and other diverse Francisella species from complex samples
Source: PLoS One. 2022 Oct 12;17(10):e0273273. doi: 10.1371/journal.pone.0273273 (PMC9555625; doi:10.1371/journal.pone.0273273)

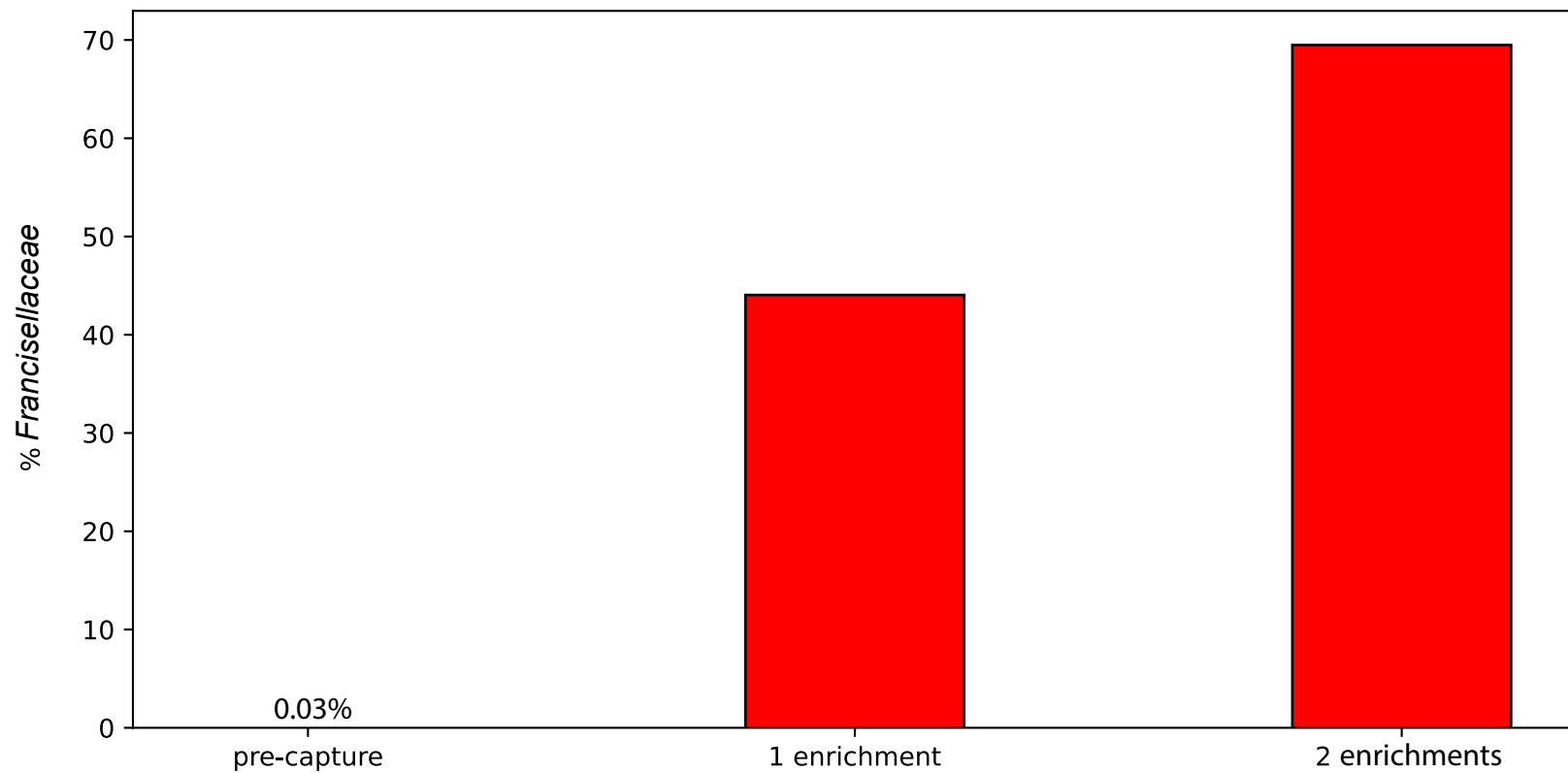

Supplement: S1 Fig — Percentage of reads classified as Francisellaceae based on Kraken2 for the spiked and unenriched dust sample, and the same sample after one and two enrichments. (PDF) [file pone.0273273.s001.pdf]

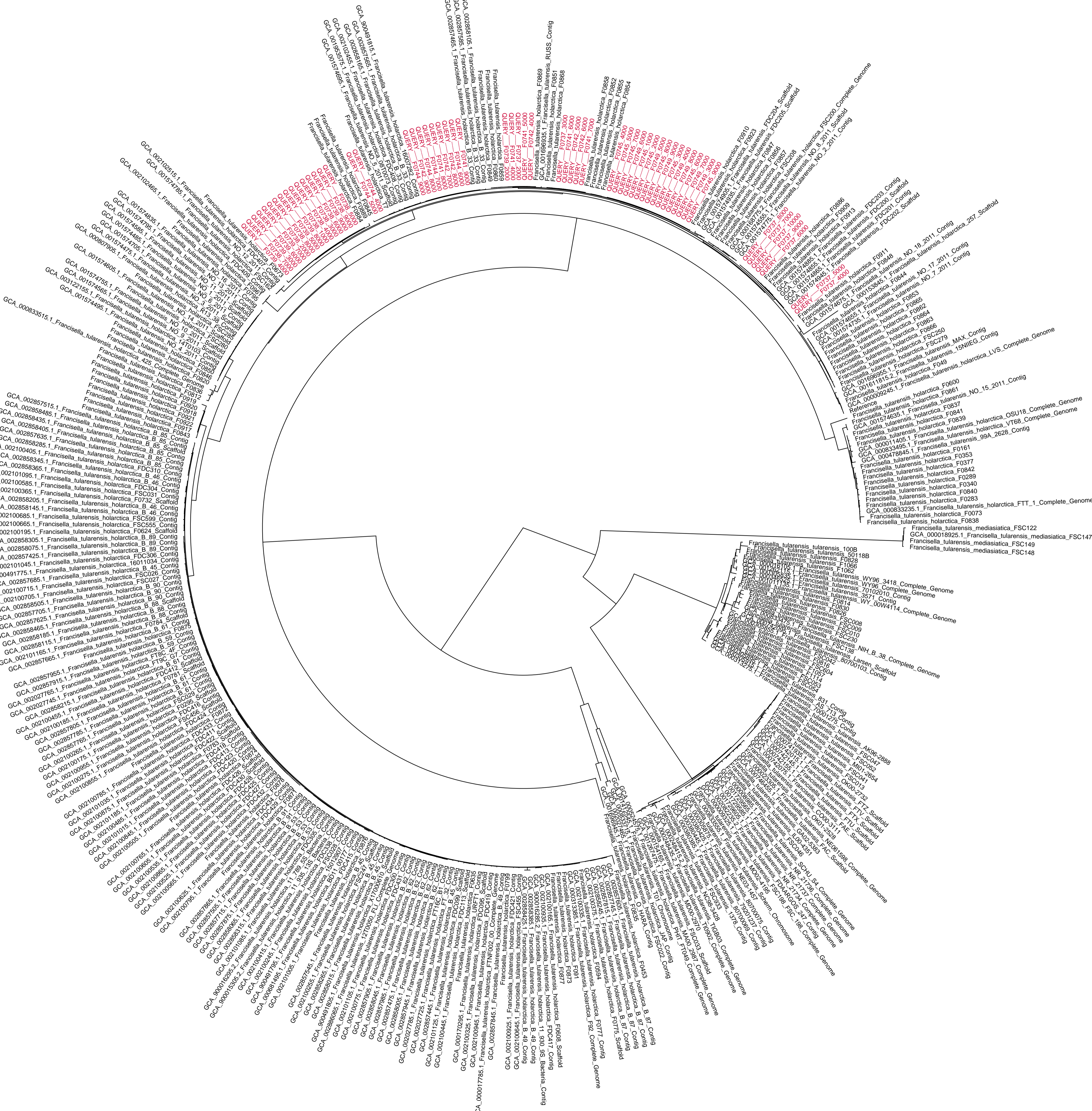

Supplement: S3 Fig — The reference phylogeny was inferred from all SNPs identified from a reference set of F. tularensis genomes (S1 Table). The query samples were inserted into the phylogeny with WG-FAST using all called SNPs. The query samples are shown in red and the number at the end of each sample name indicates the number of randomly sampled paired end reads. (PDF) [file pone.0273273.s003.pdf]
